# Recombinant Long-Acting Thioredoxin Ameliorates AKI to CKD Transition via Modulating Renal Oxidative Stress and Inflammation

**DOI:** 10.3390/ijms22115600

**Published:** 2021-05-25

**Authors:** Kento Nishida, Hiroshi Watanabe, Ryota Murata, Kai Tokumaru, Rui Fujimura, Shun Oshiro, Taisei Nagasaki, Masako Miyahisa, Yuto Hiramoto, Hiroto Nosaki, Tadashi Imafuku, Hitoshi Maeda, Masafumi Fukagawa, Toru Maruyama

**Affiliations:** 1 Department of Biopharmaceutics, Graduate School of Pharmaceutical Sciences, Kumamoto University, 5-1 Oe-honmachi, Chuo-ku, Kumamoto 862-0973, Japan; spbv8d99@gmail.com (K.N.); ryota.080708@gmail.com (R.M.); 161p1034@st.kumamoto-u.ac.jp (K.T.); murabububu@gmail.com (R.F.); 186y3004@st.kumamoto-u.ac.jp (S.O.); 191y3003@st.kumamoto-u.ac.jp (T.N.); msk07myhs@outlook.jp (M.M.); infinity72711@gmail.com (Y.H.); 184p1013@st.kumamoto-u.ac.jp (H.N.); ttt.iiii.0514@gmail.com (T.I.); maeda-h@kumamoto-u.ac.jp (H.M.); 2 Division of Nephrology, Endocrinology and Metabolism, Tokai University School of Medicine, 143 Shimo-Kasuya, Isehara 259-1193, Japan; fukagawa@tokai-u.jp

**Keywords:** thioredoxin, albumin fusion, acute kidney injury, chronic kidney disease

## Abstract

An effective strategy is highly desirable for preventing acute kidney injury (AKI) to chronic kidney disease (CKD) transition. Thioredoxin-1 (Trx), a redox-active protein that has anti-oxidative and anti-inflammatory properties, would be a candidate for this but its short half-life limits its clinical application. In this study, we examined the renoprotective effect of long-acting Trx that is comprised of human albumin and Trx (HSA-Trx) against AKI to CKD transition. AKI to CKD mice were created by renal ischemia-reperfusion (IR). From day 1 to day 14 after renal IR, the recovery of renal function was accelerated by HSA-Trx administration. On day 14, HSA-Trx reduced renal fibrosis compared with PBS treatment. At the early phase of fibrogenesis (day 7), HSA-Trx treatment suppressed renal oxidative stress, pro-inflammatory cytokine production and macrophage infiltration, thus ameliorating tubular injury and fibrosis. In addition, HSA-Trx treatment inhibited G2/M cell cycle arrest and apoptosis in renal tubular cells. While renal Trx protein levels were decreased after renal IR, the levels were recovered by HSA-Trx treatment. Together, HSA-Trx has potential for use in the treatment of AKI to CKD transition via its effects of modulating oxidative stress and inflammation.

## 1. Introduction

Acute kidney injury (AKI) is regarded as an important risk factor for the development of chronic kidney disease (CKD). When a person develops AKI, the hazard ratios for CKD onset and progression to end stage renal disease (ESRD) is increased to 8.8 and 3.1, respectively [1]. In addition, 20–50% of subjects who develop AKI have been found to transit to CKD [2]. An investigation of the increase of serum creatinine (SCr) levels within 7 days after cardiovascular surgery showed that a higher SCr contributed to the transition to CKD, suggesting that the severity of AKI represent a risk of this transition [3]. Based on the above findings, “AKI to CKD transition” is now recognized as a clinical problem, and preventive strategies are currently needed.

The mechanism responsible for the transition from AKI to CKD could involve the prolonged oxidative stress and inflammatory response induced by renal ischemia reperfusion [4,5,6]. During the normal repair of tubular cells, the damaged tubular cells are removed by surviving tubular cells or infiltrating macrophages [4,5,6]. The undamaged tubular cells proliferate to complement the missing cells caused by tubular cell death, thus allowing tubule regeneration could be completed [4,5,6]. On the other hand, when prolonged oxidative stress and inflammatory response are induced, cell death or G2/M cell cycle arrest occurs, which leads to the suppression of this cell proliferation for tubular regeneration. The suppression of tubular regeneration is accompanied by the sustained activation of infiltrating macrophages. At that time, tubular cells in a cell cycle arrest state and activated macrophages produce reactive oxygen species (ROS), inflammatory cytokines and fibrosis promoting factor (TGF-β etc). These factors induce an epithelial-mesenchymal transition in which differentiation of tubular cells or fibroblasts to myofibroblasts occurs [7]. Increased myofibroblasts produce collagen which accumulates in the extracellular matrix in the tubulointerstitium, ultimately resulting in the development of fibrosis, a common finding of CKD. These findings suggest that a strategy involving the sustainable control of oxidative stress and inflammation could be a novel therapeutic strategy for inhibiting the AKI to CKD transition.

It was recently reported that the Nrf2 activator that induces endogenous anti-oxidants and anti-inflammatory substances suppressed AKI [8]. These studies indicated that anti-oxidative and anti-inflammatory therapies against AKI might be efficacious and also suggested that the active substances are present in the Nrf2 target gene [8]. Among the Nrf2 target genes, we focused on thioredoxin (Trx), a molecule that has both anti-oxidative and anti-inflammatory activities. The renal levels of Trx are decreased during AKI [9], while Trx transgenic mice had a renoprotective effect against ischemic AKI [10]. It therefore follows that Trx would have great potential as a therapeutic drug against AKI to CKD transition. However, the molecular weight of Trx is as low as 12 kDa, and, as a result, it has a very short plasma elimination half-life in mice, about 1 hr [11]. This is a bottleneck for clinical applications. We recently developed a recombinant albumin-thioredoxin fusion protein (HSA-Trx), in which Trx was fused with human serum albumin (HSA). This product has a substantially higher blood retention and therefore a sustained Trx activity [12,13,14,15,16,17,18,19,20]. The elimination half-life of HSA-Trx in plasma was determined to be more than 10 times that of Trx, thus confirming that this albumin fusion technology produced a product with an improved Trx retention in the blood circulation [13].

In this study, we examined the therapeutic impact of HSA-Trx against the renal IR-induced AKI to CKD transition in model mice and then clarified its anti-oxidative and anti-inflammatory effect in this model.

## 2. Results

### 2.1. Evaluation of AKI to CKD Transition Model Mice

For examining the AKI to CKD transition, a renal ischemia reperfusion injury (IRI) model was used as the AKI to CKD model [21]. In this study, renal injury was induced by completely clamping both renal arteries and veins of mice, and reperfusion was induced 35 min later (Supplementary Figure S1A) [22]. Renal function markers such as blood urea nitrogen (BUN), SCr and creatinine clearance (Ccr) on days 1, 7 and 14 after renal IR treatment showed that, after day 1 post-renal IR, the BUN, SCr and Ccr levels gradually approached the baseline level (sham group) over time, and on day 14 after renal IR, renal function was completely restored to sham group (Supplementary Figure S1B–D). Renal Periodic Acid Schiff (PAS) staining and Masson’s trichrome staining were performed on day 14 after renal IR. As a result, tubule dilation, the increase of the interstitial area and the increase in the cell nuclei in the interstitium were confirmed in the PAS staining images (Supplementary Figure S1E). Masson’s trichrome staining also provided evidence that collagen accumulation occurred (renal fibrosis) in the renal interstitium of the renal IR-treated group (Supplementary Figure S1F). These data suggest that the renal IR-mice can be used as an AKI to CKD transition model.

### 2.2. Effects of HSA-Trx on Renal Function and Renal Tissue Damage during the AKI to CKD Transition

**Figure 1:**
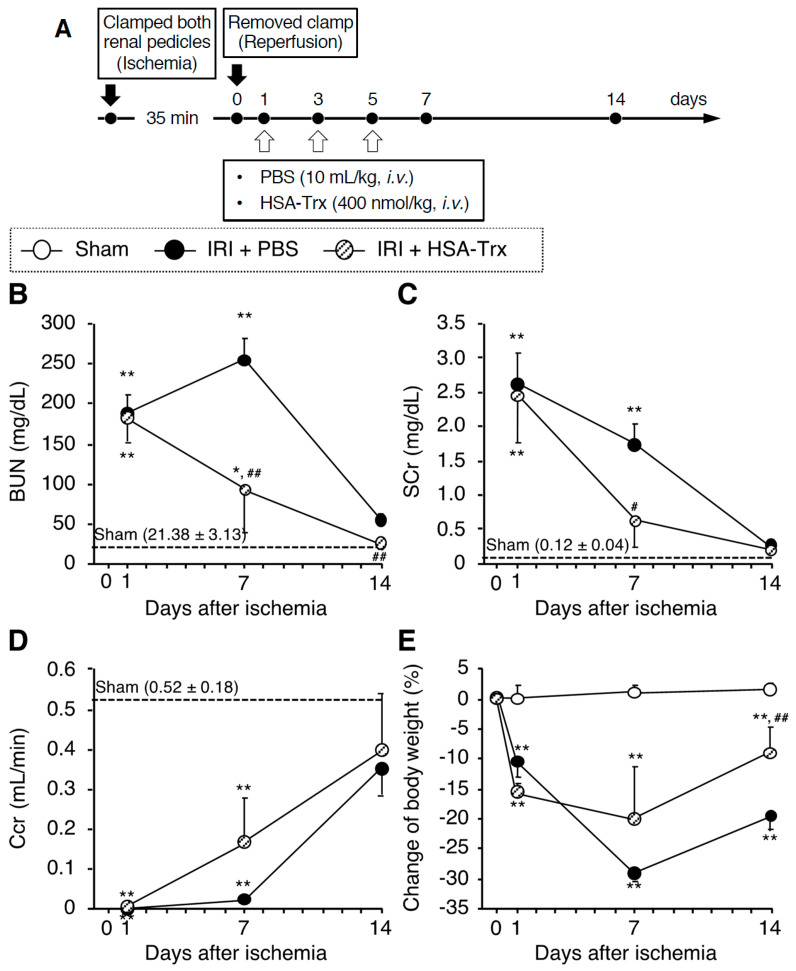

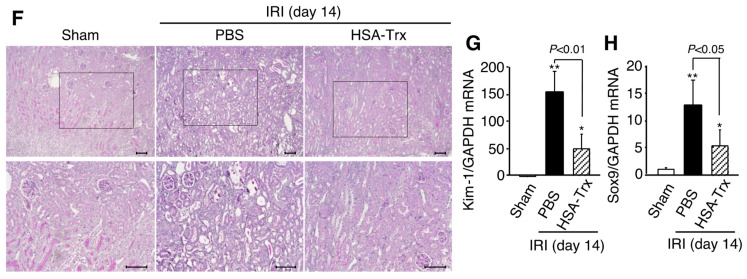
Renoprotective effect of HSA-Trx against AKI to CKD transition. (**A**) Experimental protocol for the effective evaluation of HSA-Trx on renal IR-induced AKI to CKD transition model mice: The mouse model of AKI to CKD transition was induced by renal IR where both renal pedicles were clamped for 35 min. HSA-Trx (400 nmol/kg) was administered intravenously on 1, 3 and 5 days after renal IR. An equivalent amount of PBS (10 mL/kg) was administered to the sham operation group and the renal-IR group. The mice were sacrificed 14 days after IR. (**B**) Blood urea nitrogen (BUN), (**C**) serum creatinine (SCr) and (**D**) creatinine clearance (Ccr) were measured at 1, 7 and 14 days after renal IR. (**E**) Percent change in body weight from the baseline (before renal IR). Data are expressed as the mean ±SD (*n* = 5). * *p* < 0.05, ** *p* < 0.01 compared with sham mice at each time point. ^#^ *p* < 0.05, ^##^ *p* < 0.01 compared with renal IR-mice administered with PBS at each time point. Effect of HSA-Trx against renal tubular damage in renal IR-treated mice 14 days after IR: (**F**) Representative photomicrographs of Periodic Acid Schiff (PAS)-stained kidney sections are shown for 14 days after renal IR. Lower panels are an enlarged image of the upper panel. Original magnifications: ×200 (upper panels); ×400 (lower panels). Scale bars represent 100 μm. mRNA expression of (**G**) Kim-1 and (H) Sox9 in kidney on 14 days after renal IR were determined by real-time PCR. Data are expressed as means ±SD (*n* = 5). * *p* < 0.05, ** *p* < 0.01 compared with sham mice.

The administration schedule is shown in Figure 1A. The effect of HSA-Trx on renal function was examined in initial experiments. As shown in Figure 1B–D, the recovery of renal function was enhanced in the HSA-Trx-administered group compared to the PBS-administered group on day 7 after the renal IR treatment. In addition, on day 7, the HSA-Trx administration tended to suppress body weight loss, and showed a significant recovery on day 14 compared to the PBS administration group (Figure 1E). In addition, the increased kidney to body weight ratio on day 14 in the PBS administration group was significantly suppressed by the HSA-Trx administration (data not shown). These results suggest that the administration of HSA-Trx promotes renal function recovery after the renal IR treatment.

Renal tissue damage was evaluated by PAS staining on day 14 after the renal IR treatment. The renal tubular dilation and abnormal findings in the interstitial region caused by the renal IR treatment were attenuated in the HSA-Trx administration group (Figure 1F). The mRNA expression of Kim-1 and Sox9 (tubular injury markers), which increase in response to tubular cell injury, was also evaluated in the renal tissues. In the PBS-administered group, mRNA expression levels of Kim-1 and Sox9 remained high due to incomplete tissue repair after the onset of AKI (Figure 1G,H). On the other hand, the mRNA expression of both genes was significantly suppressed in the HSA-Trx administration group compared to the PBS administration group.

### 2.3. Effect of HSA-Trx on Renal Fibrosis

**Figure 2:**
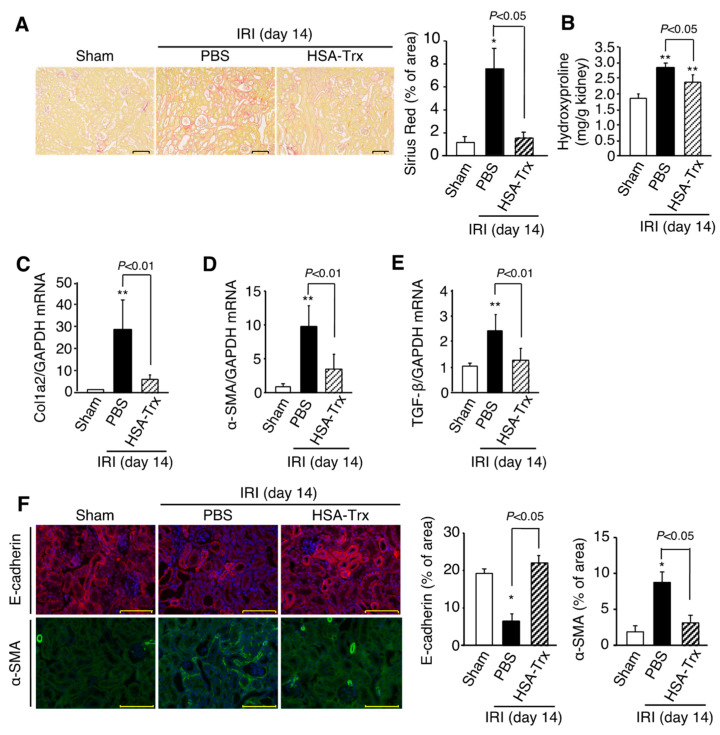
Effect of HSA-Trx against renal fibrosis and the epithelial-mesenchymal transition in renal IR-treated mice on 14 days after IR. (**A**) Representative photomicrographs and quantification of Sirius red-stained kidney sections are shown on 14 days after renal IR. Original magnifications: ×200. Scale bars represent 100 μm. (**B**) Hydroxyproline content in kidney were measured on 14 days after renal IR. (**C**) mRNA expression of (C) Col1a2, (**D**) α-SMA (α-smooth muscle actin) and (**E**) TGF-β in kidney on 14 days after renal IR were determined by real-time PCR. Effect of HSA-Trx against epithelial-mesenchymal transition (EMT) in renal IR-treated mice on 14 days after IR: (**F**) Representative photomicrographs and quantification of immunostaining of renal E-cadherin and α-SMA are shown on 14 days after renal IR. Cells were also treated with DAPI (blue) in E-cadherin and α-SMA immunostaining. Original magnifications: ×400. Scale bars represent 100 μm. Data are expressed as the mean ±SD (*n* = 5). * *p* < 0.05, ** *p* < 0.01 compared with sham mice.

On day 14 after renal IR treatment, histological evaluations were performed using Sirius red staining to evaluate renal fibrosis. As a result, fibrosis formation was observed in the PBS administration group, and the amount of hydroxyproline, the expression of Col1a2, α-SMA and TGF-β mRNA were correspondingly increased (Figure 2A–E). However, such fibrosis formation was suppressed by the HSA-Trx administration.

In addition, the expression of E-cadherin, an epithelial cell marker that was highly expressed in tubules of the sham group, was decreased in the PBS-treated group (Figure 2F). In contrast, the expression of the myofibroblast marker α-SMA, which was barely expressed in the interstitial region of the sham group, was increased in the interstitial region of the PBS-treated group (Figure 2D,F). In the HSA-Trx administration group, E-cadherin expression was recovered, and α-SMA expression were suppressed (Figure 2F), indicating that HSA-Trx has anti-renal fibrotic effects.

### 2.4. Effect of HSA-Trx on Oxidative Stress and Inflammation in the Kidney

To elucidate the mechanism by which HSA-Trx prevents the AKI to CKD transition, we focused on the renal oxidative stress and inflammation. Since HSA-Trx administration resulted in the recovery of renal function from day 1 to day 7 after renal IR treatment, this early recovery from AKI could affect renal outcome. Therefore, on day 7 after the renal IR treatment, in which the difference in renal function was observed by HSA-Trx administration, was set as the time for the evaluation, and subsequent studies were performed.

**Figure 3:**
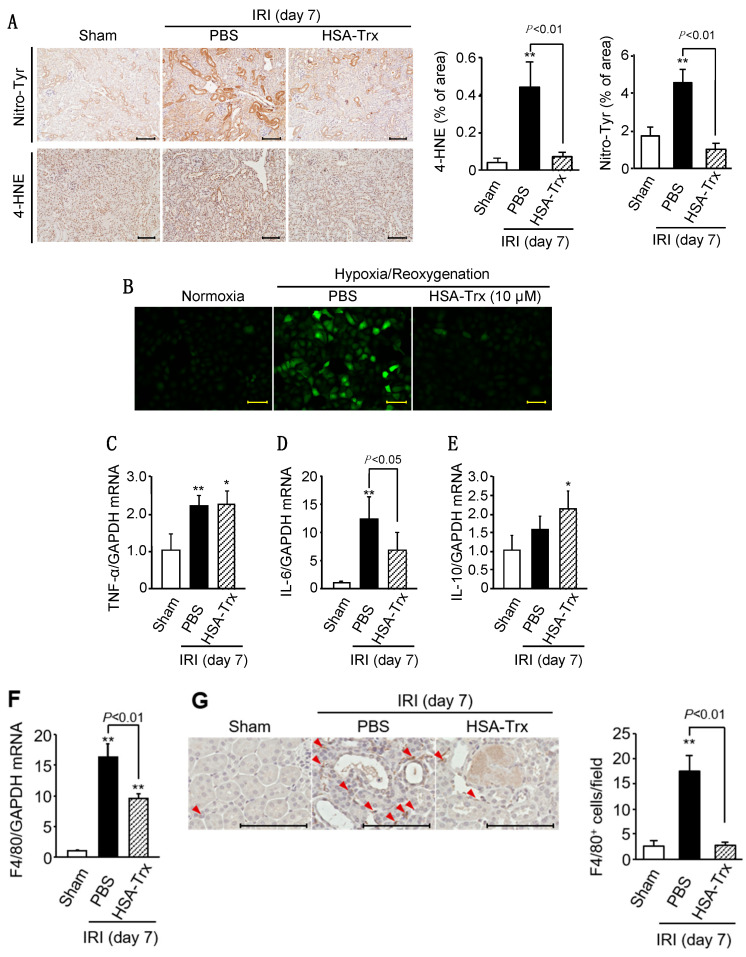
Effect of HSA-Trx on renal oxidative stress and inflammation. (**A**) Effect of HSA-Trx on renal oxidative stress in renal IR-treated mice on 7 days after renal IR: Representative photomicrographs and quantification of immunostaining of renal Nitro-Tyr (nitrotyrosine) and 4-HNE (4-hydroxynonenal) are shown on 7 days after renal IR. Original magnifications: ×200. Scale bars represent 100 μm. (**B**) Effect of HSA-Trx on cellular ROS level in hypoxia/reoxygenation-treated HK-2 cell: Cellular ROS level in HK-2 cells was detected by CM-H_2_DCFDA, an ROS-sensitive fluorescent dye. HK-2 cells were exposed to 0.1% O_2_ hypoxia (Anaeropack^®^: Mitsubishi Gas Chemical Co. Inc., Tokyo, Japan) for 24 hr in the presence or absence of 10 μM HSA-Trx, followed by reoxygenation, and the HK-2 cells were then incubated with CM-H_2_DCFCA for 30 min. After the reaction, cells were observed using a microscope. Original magnifications: ×200. Scale bars represent 100 μm. Effect of HSA-Trx on cytokine expression and macrophage infiltration in the kidneys of renal IR-treated mice on 7 days after renal IR: mRNA expression of (**C**) TNF-α, (**D**) IL-6, (**E**) IL-10 and (**F**) F4/80 in kidney on 7 days after renal IR were determined by real-time PCR. (**G**) Representative photomicrographs and quantification of immunostaining of renal F4/80 are shown on 7 days after renal IR. F4/80-positive cells are indicated by arrowheads. Original magnifications: ×800. Scale bars represent 100 μm. Data are expressed as means ±SD (*n* = 5). * *p* < 0.05, ** *p* < 0.01 compared with sham mice.

To evaluate oxidative stress, immunostaining for oxidative stress markers Nitro-Tyr and 4-HNE was performed. As a result, a marked increase in oxidative stress markers was observed at 7 days after the IR treatment, suggesting that oxidative stress was involved in the early stage of fibrosis formation (Figure 3A). In contrast to the PBS administration group, the increased oxidative stress was suppressed in the HSA-Trx administration group (Figure 3A). We confirmed the anti-oxidative effect of HSA-Trx in vitro using human tubular cells (HK-2 cells). As a result, HSA-Trx treatment decreased intracellular ROS levels as observed in hypoxia/reoxygenation-treated HK-2 cells (Figure 3B).

Next, inflammatory cytokines and macrophages were evaluated 7 days after renal IR treatment. As a result, in the PBS-administered group, the mRNA levels of the inflammatory cytokines TNF-α and IL-6 were significantly increased compared to the sham group, while the mRNA level of the anti-inflammatory cytokine IL-10 tended to increase (Figure 3C–E). In addition, the mRNA expression level of F4/80, a macrophage marker, was significantly increased (Figure 3F), and a similar result was shown by immunostaining of F4/80 (Figure 3G). These results suggest that the renal tissue environment on day 7 after renal IR treatment can be attributed to a pro-inflammatory condition. The HSA-Trx administration did not affect TNF-α mRNA expression as observed in the PBS-treated group, but it resulted in a significant suppression of IL-6 mRNA expression. Interestingly, HSA-Trx administration also significantly increased the mRNA expression of IL-10 compared to the sham group. The decreased F4/80 mRNA expression and F4/80+ cell numbers as the result of the HSA-Trx administration suggest that HSA-Trx suppresses inflammatory status on 7 days after the renal IR treatment.

### 2.5. Effects of HSA-Trx on the Cell Cycle and Apoptosis of Tubular Cells

**Figure 4:**
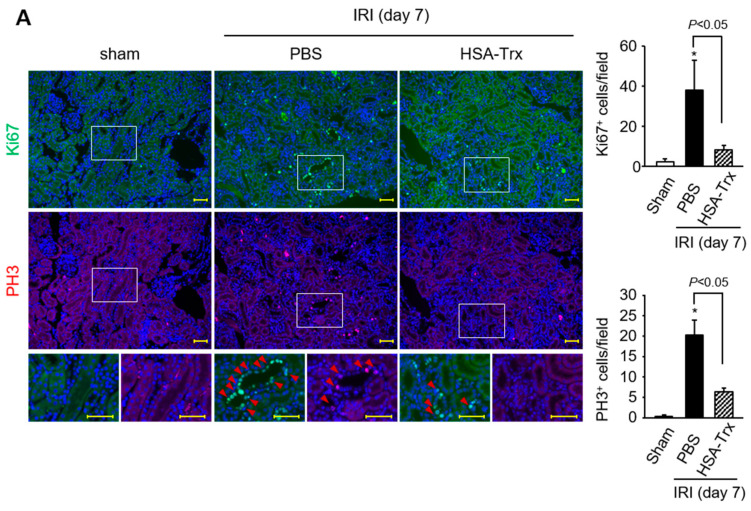

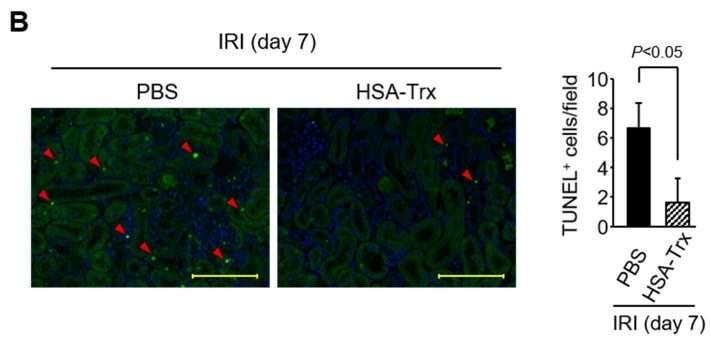
Effect of HSA-Trx on the G2/M arrest of renal tubular cells and tubular apoptosis in renal IR-treated mice at 7 days after renal IR. (**A**) Representative photomicrographs and quantification of the immunostaining of renal Ki67 (green) and PH3 (Phospho-Histone H3, red) are shown 7 days after renal IR. Cells were also treated with DAPI (blue). Serial sections of kidney were used in Ki67 and PH3 immunostaining. Ki67 or PH3-positive cells are indicated by arrowheads. Lower panels are enlarged images of the upper and middle panel. Original magnifications: ×100. Scale bars represent 100 μm. * *p* < 0.05 compared with sham mice. (**B**) Effect of HSA-Trx on renal tubular cell apoptosis in renal IR-treated mice during 7 days after renal IR: Representative photomicrographs and quantification of TUNEL-stained kidney sections are shown. TUNEL -positive cells are indicated by arrowheads. Cells were also treated with DAPI (blue). Original magnifications: ×400. Scale bars represent 100 μm.

To investigate the involvement of G2/M cell cycle arrest in this pathological model, using serial sections of kidneys collected on day 7 after the renal-IR treatment, immunostaining was performed for Ki67, a marker for the proliferative phase (G1, S, G2, M phases), and phosphorylated histone H3 (PH3), a marker for the G2/M phase immunostaining. G2/M cell cycle arrest was evaluated by regarding Ki67+ cells as being proliferating cells and PH3+ cells as being G2/M cells. As a result, the levels of Ki67+ cells and PH3+ cells were increased by renal-IR treatment but HSA-Trx administration suppressed them (Figure 4A). These data suggest that HSA-Trx administration suppressed G2/M cell cycle arrest.

The effect of HSA-Trx administration on tubular apoptosis was evaluated by TUNEL staining (Figure 4B). The findings indicated that TUNEL-positive cells in the PBS-treated group were increased on day 7 after renal IR treatment. HSA-Trx administration reduced the number of TUNEL-positive cells, indicating that HSA-Trx suppressed tubular apoptosis.

### 2.6. Endogenous Trx Expression in the AKI to CKD Transition

**Figure 5:**
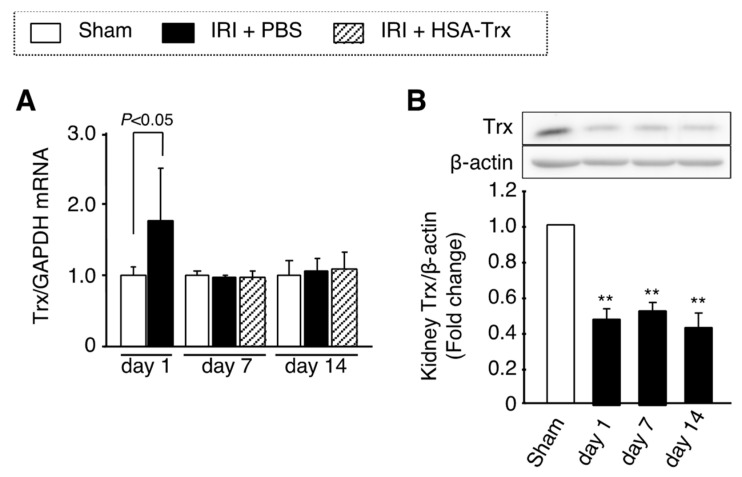

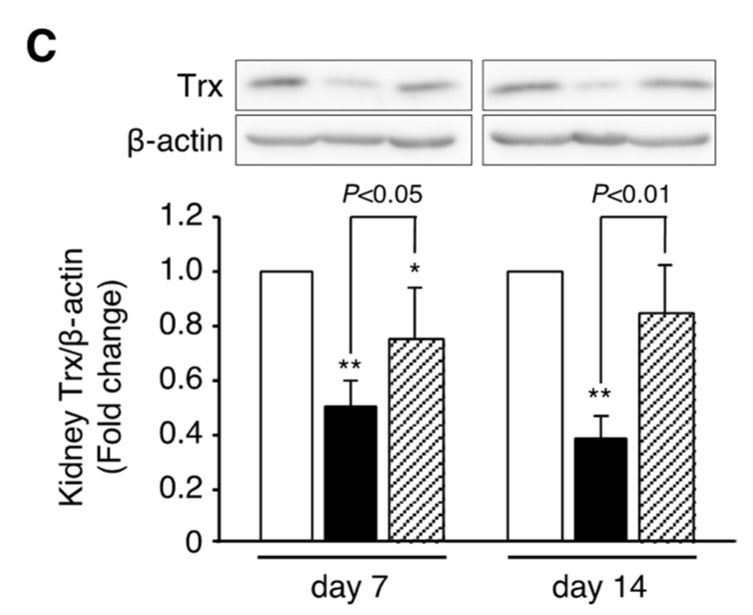
Effect of HSA-Trx on endogenous Trx expression in kidney of renal IR-treated mice. (**A**) Trx mRNA expression in kidney on 1, 7 and 14 days after renal IR were determined by real-time PCR. (**B**) Trx protein expression in kidney on 1, 7 and 14 days after renal IR, and (**C**) Trx protein expression in kidney on 7 and 14 days after renal IR with or without HSA-Trx treatment were assessed by Western blotting. The intensity of each band was quantified using the ImageJ software program and normalized against β-actin expression. Data are expressed as the mean ±SD (*n* = 4). * *p* < 0.05, ** *p* < 0.01 compared with sham mice.

The mRNA expression and protein levels of endogenous Trx in kidney tissue were evaluated by real-time PCR and Western blotting. As a result, in the PBS-administered group, the mRNA expression level of Trx showed a significant increase only on day 1 after the renal IR treatment (Figure 5A). The amount of Trx protein in renal tissue was significantly decreased from day 1 after the renal IR treatment, and this decrease was continued until day 14 (Figure 5B). Interestingly, the amount of Trx protein in the renal tissue of the HSA-Trx administration group was significantly increased compared to that for the PBS administration group, suggesting the endogenous Trx protein level in kidney tissue was sustained by HSA-Trx administration (Figure 5C). These results suggest that the administration of HSA-Trx alleviates the decrease in endogenous Trx level which could contribute to the suppression of the AKI to CKD transition.

## 3. Discussion

Recent epidemiological studies revealed that AKI is deeply involved in the development and progression of CKD. In addition, it has also been reported that the more severe tubular damage that occurs at the AKI induction contributed to prolonged kidney injury and caused CKD [6]. Therefore, the key to preventing the AKI to CKD transition is to minimize the severity of AKI to the extent possible. In this study, we demonstrated that HSA-Trx has a renoprotective effect on AKI to CKD transition using a mouse model of AKI to CKD transition induced by renal-IR.

The tubular injury observed on day 1 after the renal IR treatment was suppressed on day 7 by the HSA-Trx administration (Supplementary Figure S2A). In addition, the mRNA expressions of the tubular injury marker Kim-1 and the tubular repair marker Sox9 in renal tissue were also suppressed in the HSA-Trx-administered group (Supplementary Figure S2B,C). The expression of Kim-1 increases from the early stage of AKI, depending on the degree of tubular injury, and converges with tubular repair. However, it has been reported that the delayed or incomplete repair of tubule repair causes a prolonged increase in its expression [23]. Sox9 expression has been reported to behave similarly [24]. Based on these findings, it was suggested that HSA-Trx caused an enhanced tubular repair, leading to the suppression of tubular injury on day 7 after the renal IR treatment.

As shown in Figure 3A, the HSA-Trx administration suppressed renal oxidative stress at day 7 after the renal IR treatment. It is well known that oxidative stress associated with AKI induces inflammation and fibrosis-related signaling [25,26,27]. Since therapeutic intervention with anti-oxidants has been shown to be effective in a unilateral ureteral ligation (UUO) model [28] and remnant kidney models [29,30], it is presumed that HSA-Trx ameliorates the AKI to CKD transition by suppressing oxidative stress in renal tissue. As shown in Figure 5B,C, the renal IR treatment decreased the protein expression of endogenous Trx (day 1 to day 14) in renal tissue while the mRNA expression of endogenous Trx was increased only on day 1 after the renal IR treatment (Figure 5A). It is also known that Trx expression is induced by the activation of Nrf2, a transcription factor that is responsive to oxidative stress [31,32,33]. In addition, the intensity of oxidative stress on day 1 was higher than that on day 14 after the renal IR treatment (Supplementary Figure S3 and Figure 3A), indicating that Nrf2 activation in response to oxidative stress on day 1 after the renal-IR treatment might increase the expression of endogenous Trx mRNA. Kasuno et al. reported that Trx, which is normally localized in the entire tubule, was localized to the luminal side after a renal IR treatment, which then increased urinary excretion [9]. They also reported that the treatment of a human proximal tubular epithelial cell line with hydrogen peroxide enhanced the secretion of Trx into the culture supernatant, and treatment with an anti-oxidant, *n*-acetylcysteine, suppressed this secretion. Although the mechanism responsible for the extracellular secretion of Trx remains unclear, the above findings suggest that Trx in renal tubular epithelial cells is secreted to the luminal side by oxidative stress due to the renal-IR treatment, and, as a result, renal tubular Trx protein levels could be decreased. Interestingly, HSA-Trx administration recovered this decrease in renal Trx protein levels as observed in the PBS-treated group (Figure 5C). This result could be explained by the fact that HSA-Trx administration suppressed oxidative stress in renal tubular cells, thereby suppressing endogenous intracellular Trx loss. Unfortunately, since the anti-Trx antibody used in this study reacts with both mouse and human Trx, it is also possible that the administered HSA-Trx was partially translocated into the cells and complemented Trx in kidney tissue. Further investigations will clearly be needed to examine this issue using human or mouse-specific Trx antibodies if these antibodies could be commercially available in the future. In addition, recently, several reports have demonstrated that renin-angiotensin aldosterone system (RAAS) blockade serves for retarding the transition of AKI to CKD [34,35]. Considering the pharmacological action of Trx, Trx may not directly affect RAAS system but Trx could affect the oxidative stress, the downstream of RAAS system.

Regarding tubular injury, HSA-Trx administration suppressed G2/M cell cycle arrest (Figure 4A) and tubular apoptosis (Figure 4B). It is known that JNK signals play a central role in tubular epithelial cell damage during the development of renal fibrosis [36]. In addition, it has been reported that ASK1, which is located upstream of the JNK signal, also plays a role in promoting renal damage [37]. Inflammatory cytokines such as TNF-α and TGF-β, which are produced during CKD progression, act on tubular cells, leading to the activation of ASK1 via the intracellular production of ROS, where ASK1 is dissociated from Trx and enhances the downstream JNK signals to cause tubular cell damage. In fact, Liles et al. reported that ASK1 and JNK signaling were activated in renal-IR and UUO models, and that the administration of an ASK1 inhibitor suppressed renal injury and fibrosis via the suppression of the ASK1-JNK signal [38]. In this study, we did not examine the effect of HSA-Trx on ASK1 and JNK signaling. However, the fact that the amount of endogenous Trx protein in renal tissue was maintained as the result of the HSA-Trx administration suggests that HSA-Trx could protect tubular epithelial cells via the inhibition of ASK1-JNK signaling. Further investigations will clearly be needed in the future to solve this problem. This study demonstrated that a proof of concept for a possible therapeutic of HSA-Trx against AKI to CKD transition to be deployed in the clinic. Further studies regarding scalability, safety and treatment window etc. of HSA-Trx should be needed in the future study.

## 4. Materials and Methods

### 4.1. Expression and Purification of HSA-Trx Fusion Protein

The *Pichia* Expression Kit was purchased from Invitrogen (Carlsbad, CA, USA). The production and purification of HSA-Trx was performed as described in a previously reported method [16].

### 4.2. Mouse Model of AKI to CKD Transition

The study was carried out in compliance with the ARRIVE guidelines. C57BL/6N mice (male, 8 weeks, Japan SLC, Inc., Shizuoka, Japan) were maintained in a room under controlled temperature conditions with a 12 h light and 12 h dark cycle (light 8 am–8 pm) and freely provided with food and water. All animal experiments were conducted using procedures approved by the experimental animal ethics committee at Kumamoto University (2019-017 R1, October, 2019) and all experimental protocols were performed in accordance with the relevant guidelines and regulations. To induce AKI, both renal pedicles of mice were clamped for 35 min, as described in detail in a previous report [39,40]. After randomizing the mice on day 1, they were intravenously administered a phosphate-buffered saline (PBS) as control or the HSA-Trx fusion protein (HSA-Trx) (400 nmol/kg) on day 1, 3 and 5 after reperfusion. Details of the procedures are given in the supplemental methods.

### 4.3. Biochemical Evaluation of Blood Samples

The mean BUN and SCr levels were determined by a FUJI DRI-CHEM 7000 and DRI-CHEM slides system (FUJIFILM, Tokyo, Japan). The mean urinary creatinine level was measured by the respective assay kits (Wako Pure Chemical, Osaka, Japan). Creatinine clearance (Ccr) during the 24 h period was calculated as mL/min.

### 4.4. Histological Examination of Kidney Tissues

Harvested kidney tissues were fixed in 10% formalin neutral buffer solution for 48 hr and then embedded in paraffin. Kidney blocks were cut into 2-μm sections and then subjected to PAS staining for morphologic analysis, Sirius red staining for fibrosis, TUNEL staining for cell apoptosis, immunohistostaining of nitrotyrosine (Nitro-Tyr) and 4-hydroxynonenal (4-HNE) for oxidative stress, immunohistostaining of E-cadherin and α-smooth muscle actin (α-SMA) for epithelial-mesenchymal transition (EMT), immunohistostaining of F4/80 for macrophage infiltration and immunohistostaining of Ki67 and phospho histone H3 (PH3) for cell cycle. Images and quantification analyses were performed using a Keyence BZ-X710 microscope. (Keyence, Osaka, Japan). Details of the procedures are given in the supplemental methods.

### 4.5. Measurement of Renal Hydroxyproline

Renal hydroxyproline assays were performed as described previously [41].

### 4.6. mRNA Expression Analysis

Quantitative RT-PCR was performed [42]. The details of the procedure are described in the supplemental methods (Supplementary Table S1).

### 4.7. Western Blot Analysis of Trx Expression in Kidney Tissue

For evaluation of Trx protein expression in the kidney of renal IR-treated mice, Western blotting was performed. Details of the procedure are given in the supplemental methods.

### 4.8. Statistical Analyses

Data from animal and cell studies were compared by analysis of variance followed by Tukey’s multiple comparison. The means for two group data were compared by the unpaired t-test. All results are expressed as the mean ±SD of the indicated experiments. A *p* value < 0.05 was considered to be statistically significant.

## 5. Conclusions

**Figure 6:**
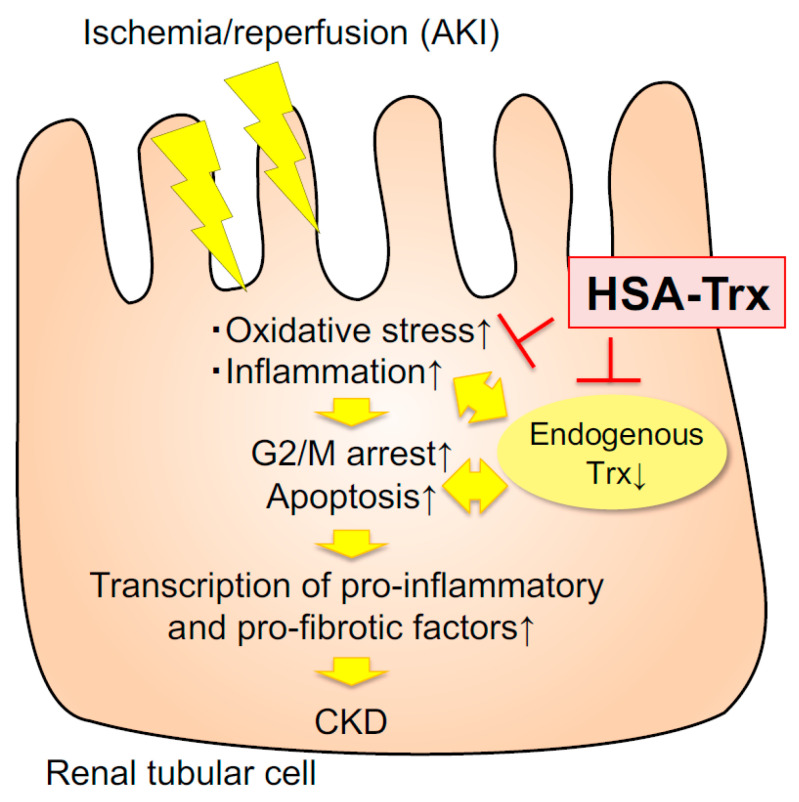
Schematic diagram of the effect of HSA-Trx on the AKI to CKD transition. AKI increased (↑) oxidative stress and inflammation, then decreased (↓) endogenous Trx.

In this study, we demonstrated that HSA-Trx efficiently promoted tubular repair by reducing the level of oxidative stress and the inflammatory environment in AKI, resulting in the suppression of CKD transition (Figure 6). Therefore, HSA-Trx could be a new AKI therapeutic agent that is effective not only for prevention and treatment of AKI but also for the AKI to CKD transition.

## Data Availability

Data are contained within the article.

## Supplementary Materials

Supplementary materials can be found at https://www.mdpi.com/article/10.3390/ijms22115600/s1.

## Abbreviations

AKI	acute kidney injury
CKD	chronic kidney disease
HSA	human serum albumin
HSA-Trx	human serum albumin-thioredoxin fusion protein
Trx	thioredoxin
