# Supplementary material for: Recombinant Long-Acting Thioredoxin Ameliorates AKI to CKD Transition via Modulating Renal Oxidative Stress and Inflammation
Source: Int J Mol Sci. 2021 May 25;22(11):5600. doi: 10.3390/ijms22115600 (PMC8199127; doi:10.3390/ijms22115600)
Supplement: Supplementary file 1 [file ijms-22-05600-s001.zip › ijms-1150546-SI.pdf]

Supplementary Materials

Recombinant Long-Acting Thioredoxin Ameliorates AKI to CKD Transition via Modulating Renal Oxidative Stress and Inflammation

Kento Nishida [1,†], Hiroshi Watanabe [1,*,†], Ryota Murata [1], Kai Tokumaru [1], Rui Fujimura [1], Shun Oshiro [1], Taisei Nagasaki [1], Masako Miyahisa [1], Yuto Hiramoto [1], Hiroto Nosaki [1], Tadashi Imafuku [1], Hitoshi Maeda [1], Masafumi Fukagawa [2] and Toru Maruyama [1,*]

1 Department of Biopharmaceutics, Graduate School of Pharmaceutical Sciences, Kumamoto University, 5-1, Oe-honmachi, Chuo-ku, Kumamoto 862-0973, Japan; spbv8d99@gmail.com (K.N.); ryota.080708@gmail.com (R.M.); 161p1034@st.kumamoto-u.ac.jp (K.T.): murabububu@gmail.com (R.F.); 186y3004@st.kumamoto-u.ac.jp (S.O.); 191y3003@st.kumamoto-u.ac.jp (T.N.); msk07myhs@outlook.jp (M.M.); infinity72711@gmail.com (Y.H.); 184p1013@st.kumamoto-u.ac.jp (H.N.); ttt.iiii.0514@gmail.com (T.I.); maeda-h@kumamoto-u.ac.jp (H.M.)

2 Division of Nephrology, Endocrinology and Metabolism, Tokai University School of Medicine, 143 Shimo-Kasuya, Isehara 259-1193, Japan; fukagawa@tokai-u.jp

* Correspondence: hnabe@kumamoto-u.ac.jp (H.W.); tomaru@gpo.kumamoto-u.ac.jp (T.M.); Tel.: +81-96-371-4855 (H.W.); +81-96-371-4150 (T.M.); Fax: +81-96-371-4855 (H.W.); +81-96-371-4153 (T.M.)

† These authors contributed equally to this work.

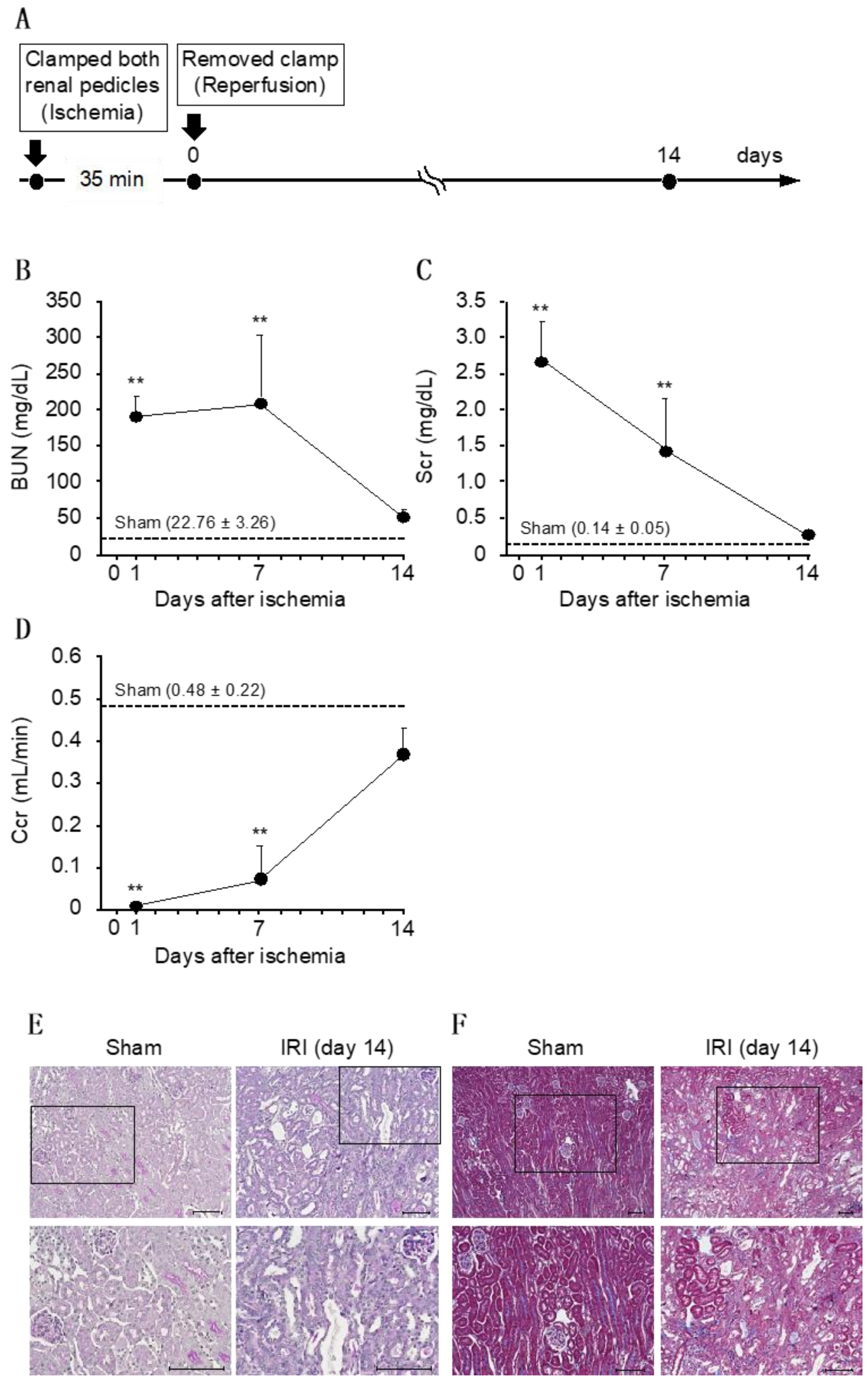

Supplemental Figure S1. Evaluation of renal function and renal injury in renal IR-treated mice at 14 days after IR. (**A**) Experimental protocol for evaluating the validity of using AKI to CKD transition model mice: The mouse model of an AKI to CKD transition was induced by renal ischemia-reperfusion injury (IRI) where both renal pedicles were clamped for 35 min. The mice were sacrificed on 14 days after IR. (**B**) Blood urea nitrogen (BUN), (**C**) serum creatinine (SCr) and (**D**) creatinine clearance (Ccr) were measured at 1, 7 and 14 days after renal IR. Data are expressed as means ±SD (n = 5). ** $p < 0.01$ compared with sham mice at each time point. Evaluation of renal histological alterations and fibrosis on 14 days after IR: Representative photomicrographs of (**E**) PAS-stained and (**F**) Masson's trichrome-stained kidney sections are shown. Lower panels are an enlarged image of the upper panel. Original magnifications: (**E**) ×200 (upper panels), ×400 (lower panels); (**F**) ×100 (upper panels), ×200 (lower panels). Scale bars represent 100 μm.

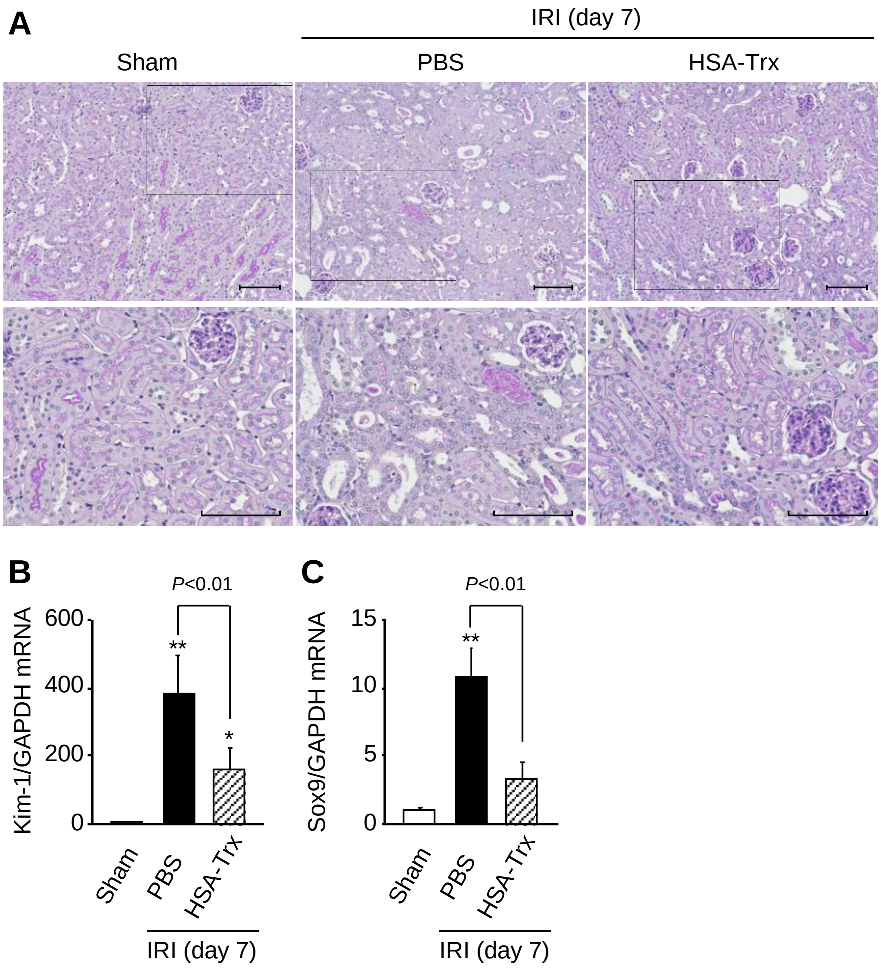

Supplemental Figure S2. Effect of HSA-Trx on renal tubular damage in renal IR-treated mice on 7 days after IR: (**A**) Representative photomicrographs of PAS-stained kidney sections on 7 days after renal IR are shown. Lower panels are an enlarged image of the upper panel. Original magnifications: ×200 (upper panels); ×400 (lower panels). Scale bars represent 100 μm. mRNA expression of (**B**) Kim-1 and (C) Sox9 in kidney on 7 days after renal IR were determined by real-time PCR. Data are expressed as the mean ±SD ($n = 5$). * $p < 0.05$, ** $p < 0.01$ compared with sham mice.

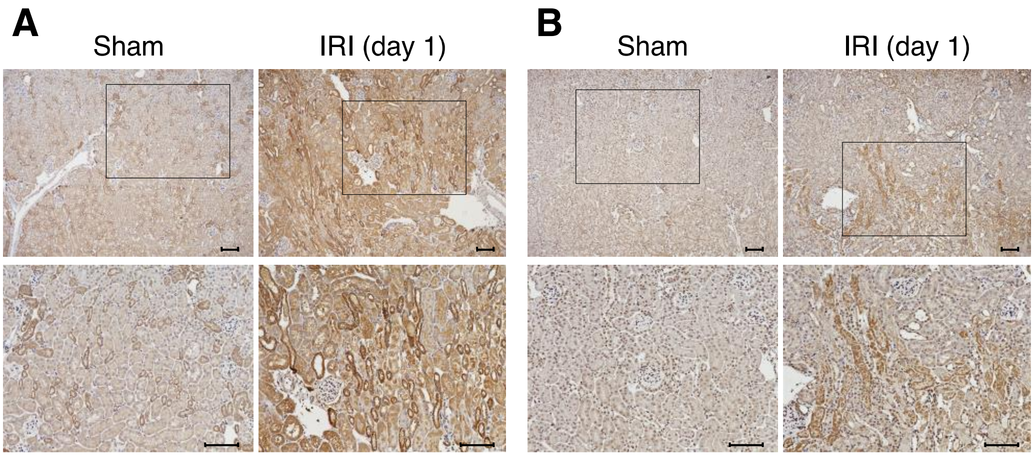

Supplemental Figure S3. Evaluation of renal oxidative damage in renal IR-treated mice at 24 h after IR: Representative photomicrographs of immunostaining of renal (**A**) Nitro-Tyr (nitrotyrosine) and (**B**) 4-HNE (4-hydroxynonenal) at 24 h after renal IR are shown. Lower panels are an enlarged image of the upper panel. Original magnifications: ×100 (upper panels); ×200 (lower panels). Scale bars represent 100 μm.

Supplemental Table S1. Primers used in quantitaive RT-PCR.

Target Gene	Forward (5′→3′)	Reverse (5′→3′)
Kim-1	TCCACACATGTACCAACATCAA	GTCACAGTGCCATTCCAGTC
Sox9	GGAAGTCGGTGAAGAACGGA	AGATTGCCCAGAGTGCTCG
IL-6	TCTCTGCAAGAGACTTCCATCC	AGACAGGTCTGTTGGGAGTG
TNF-α	CATGAGCACAGAAAGCATGATCCG	AAGCAGGAATGAGAAGAGGCTGAG
IL-10	GGACAACATACTGCTAACCGACTC	AAAATCACTCTTCACCTGCTCCAC
F4/80	CATAAGCTGGGCAAGTGGTA	GGATGTACAGATGGGGGATG
Col1a2	CACCCCAGCGAAGAACTCATA	GCCACCATTGATAGTCTCTCCTAAC
α-SMA	AGCCATCTTTCATTGGGATGG	CCCCTGACAGGACGTTGTTA
TGF-β	GGATACCAACTATTGCTTCAGCTCC	AGGCTCCAAATATAGGGGCAGGGTC
Trx1	CAAGCTTGTCGTGGTGGACTTC	GGTCGGCATGCATTTGACTTC
GAPDH	AACTTTGGCATTGTGGAAGG	ACACATTGGGGGTAGGAACA

Expanded Material and Methods:

Mouse model of acute kidney injury (AKI) to chronic kidney disease (CKD) transition

All animal experiments were performed according to the guidelines, principles, and procedures for the care and use of laboratory animals of Kumamoto University. C57BL/6N mice (male, 8 weeks, Japan SLC Inc., Shizuoka, Japan) were maintained in a temperature-controlled room with a 12 hr dark/light cycle and *ad libitum* access to food and water. To induce AKI, both renal pedicles were clamped for 35 min, as described in detail previously[1,2]. After randomizing the mice by blood urea nitrogen (BUN) and serum creatinine (SCr) on day 1, the mice were intravenously administered phosphate-buffered saline (PBS) as control or HSA-Trx fusion protein (HSA-Trx) (400 nmol/kg) on day 1, 3 and 5 days after reperfusion. On day 1, 7 and 14 after reperfusion, urine samples were collected by metabolic cages for 24 hr and were used for the determination urinary creatinine excretion. After collecting blood samples for the determination of BUN and SCr, the mice were sacrificed under anesthesia at each time following reperfusion. The kidneys were harvested and bisected in the equatorial plane, then parts of the right kidney were homogenized for quantitative RT-PCR and Western blotting, and parts of the left kidney were used for hydroxyproline assay and fixed in 10% formalin neutral buffer solution for routine histological examination, respectively. The survival rate throughout the experimental period was 100%.

Evaluation of apoptosis

For the evaluation of cell apoptosis, terminal deoxynucleotidyl transferase-mediated dUTP nick-end labeling (TUNEL) staining were performed using an *In situ* cell death detection kit, Fluorescein (Roche, Basel, Switzerland). DAPI (Dojin Chemical, Kumamoto, Japan) was also used to detect nuclei in tissue sections. After the reaction, slides were observed using a microscope (BZ-X700; Keyence, Osaka, Japan).

Evaluation of oxidative stress and macrophage infiltration by immunohistochemistry

Kidney sections were subjected to immunohistochemistry (nitrotyrosine (Nitro-Tyr), 4-hydroxynonenal (4-HNE) and F4/80). First, antigen retrieval and solubilization of the tissue sections conducted, followed by treatment with 30% H_2O_2/methanol solutions at R/T for 30 min in order to inhibit endogenous peroxidase. The tissue sections were reacted with the primary antibody (Rabbit anti-nitrotyrosine polyclonal antibody; Chemicon International, Temecula, CA; cat#: AB5411, diluted 1:50, Rabbit anti-4-hydroxynonenal polyclonal antibody; Bioss, MA, cat#: bs-6313R, diluted 1:100 or Rat anti-F4/80 monoclonal antibody; Thermo Fisher, MA, cat#:14-4801-02, diluted 1:50) overnight at 4°C. The tissue sections were then reacted with Histofine Simple Stain MAX PO (Rabbit or Rat) (Nichirei Biosciences, Tokyo, Japan) at R/T for 30 min, followed by reaction with DAB solution at R/T for 3 min. After the reactions, slides were observed using a microscope (BZ-X700; Keyence, Osaka, Japan).

Evaluation of epithelial-mesenchymal transition (EMT) and cell cycle by immunohistochemistry

Kidney sections were subjected to immunohistochemistry (E-cadherin, α-smooth muscle actin (α-SMA), Ki67 and phospho histone H3 (PH3)). First, antigen retrieval was conducted by HistoVT One (Nacalai Tesque, Kyoto, Japan) at 95°C for 30 min. A solution containing 50 mM Tris/HCl + 0.1% Tween-20 (T-TB) was then used to solubilize the tissue sections, followed by blocking with Block Ace (Dainippon Pharmaceutics, Osaka, Japan) at room temperature (R/T) for 15 min. The tissue sections were reacted with the primary antibody (goat anti-E-cadherin polyclonal antibody; R&D System, MN; cat#: AF748, diluted 1:100, rabbit anti-alpha smooth muscle actin polyclonal antibody, Abcam, Cambridge, UK; cat#: ab5694, diluted 1:100, rabbit anti-Ki67 monoclonal antibody, CST Japan, Tokyo, Japan; cat#: 12202, diluted 1:400 or rabbit anti-phospho histone H3 antibody, Invitrogen, Carlsbad, CA, cat#: PA5-17869, diluted 1:200) overnight at 4°C. The tissue sections were then washed with T-TB, followed by reaction with the secondary antibody (Alexa Fluor 647 donkey anti-goat IgG (H + L), cat#: A-21447, Alexa Fluor 488 goat anti-rabbit IgG (H + L), cat#: A-11008 and Alexa Fluor 647 chicken anti-rabbit IgG (H + L), cat#: A-21443; Invitrogen, Carlsbad, CA diluted 1:200) at R/T for 1.5 hr. DAPI (Dojin Chemical, Kumamoto, Japan) was also used to detect nuclei in tissue sections. After the reaction, slides were observed using a microscope (BZ-X700; Keyence, Osaka, Japan).

mRNA expression analysis

Quantitative RT-PCR analysis was performed as described in a previous report[3]. In a typical run, total RNA was extracted using RNAiso PLUS (TaKaRa Bio Inc., Shiga, Japan) according to the manufacturer's protocol. The concentration and the purity of the RNA extract was determined based on the absorbance at 260 and 280 nm. The cDNA was synthesized using the PrimeScript RT master mix (TaKaRa Bio Inc.). Quantitative RT-PCR analysis was performed in an iCycler thermal cycler (Bio-Rad, Hercules, CA) with an iQ5 qRT-PCR detection system attached (Bio-Rad) using SYBR Premix Ex TaqII (TaKaRa Bio Inc.). Polymerase chain reaction amplifications were performed under the following conditions: 95°C for 3 min, for 40 cycles at 95°C for 10 s (denaturation step), at 60°C for 1 min (annealing/extension steps). The sequences of the oligonucleotide primers used are shown in Supplemental Table. The threshold cycle (Ct) values for each gene amplification were normalized by subtracting the Ct value calculated for the internal standard.

Western blot analysis of thioredoxin (Trx) expression in kidney tissue

Western blotting of Trx in kidney was performed as described in a previous report[4]. The supernatant of homogenized kidney tissue was separated by SDS-PAGE and transferred to polyvinylidene difluoride membranes (Immobilon-P; Millipore,Billerica, MA) by wet electroblotting. The membranes were washed three times with Tris-buffered saline containing 0.1% Tween 20 (TBS-T) and then blocked with 5% skimmed milk in TBS-T for 1 hr at R/T. The membranes were washed three times with TBS-T and then incubated overnight at 4°C with the primary antibody (Rabbit anti-Thioredoxin 1 monoclonal antibody; CST Japan, Tokyo, Japan, cat#: 2429, diluted 1:2000 and monoclonal anti-◎-actin; Sigma-Aldrich, St Louis, MO; cat#: A5411, diluted 1:5000) in TBS-T. The membranes were washed three times with TBS-T and then incubated with the secondary antibody (mouse anti-rabbit IgG-HRP; Santa Cruz Biotechnology, cat#: sc-2357, diluted 1:2000 and m-IgGκ BP-HRP; Santa Cruz Biotechnology, cat#: sc-516102, diluted 1:5000) in TBS-T for 1 hr at R/T. The membranes were washed three times with TBS-T, and the immunoblots were visualized using the SuperSignal West Pico chemiluminescent substrate (Pierce Biotechnology Inc., Rockford, IL) or ImmunoStar LD (FUJIFILM, Tokyo, Japan) with LAS-4000EPUVmini (GE Healthcare, Amersham, UK). The intensity of each band was quantified using the ImageJ software.

Supplemental References

1. Hassoun, H. T. *et al.* Ischemic acute kidney injury induces a distant organ functional and genomic response distinguishable from bilateral nephrectomy. *Am J Physiol Renal Physiol* **293**, F30-40, doi:10.1152/ajprenal.00023.2007 (2007).
2. Hassoun, H. T. *et al.* Kidney ischemia-reperfusion injury induces caspase-dependent pulmonary apoptosis. *Am J Physiol Renal Physiol* **297**, F125-137, doi:10.1152/ajprenal.90666.2008 (2009).

3. Enoki, Y. *et al.* Potential therapeutic interventions for chronic kidney disease-associated sarcopenia via indoxyl sulfate-induced mitochondrial dysfunction. *J Cachexia Sarcopenia Muscle* **8**, 735-747, doi:10.1002/jcsm.12202 (2017).
4. Nishida, K. *et al.* Renoprotective effect of long acting thioredoxin by modulating oxidative stress and macrophage migration inhibitory factor against rhabdomyolysis-associated acute kidney injury. *Sci Rep* **5**, 14471, doi:10.1038/srep14471 (2015).
